# Real-world experience: a retrospective pediatric chart review to determine why patients and caregivers discontinue oral immunotherapy

**DOI:** 10.1186/s13223-024-00912-9

**Published:** 2024-10-15

**Authors:** Amy A. Plessis, Scott B. Cameron, Rosemary Invik, Mariam Hanna, Douglas P. Mack, Victoria E. Cook

**Affiliations:** 1 Division of Allergy, Department of Pediatrics, University of British Columbia, Vancouver, BC Canada; 2 Division of Immunology, Department of Pediatrics, University of British Columbia, Vancouver, BC Canada; 3 Community Allergy Clinic, Victoria, BC Canada; 4 Department of Pediatrics, Faculty of Medicine, University of British Columbia Island Medical Program, Victoria, BC Canada; 5 Department of Pediatrics, McMaster University, Hamilton, ON Canada

**Keywords:** Oral immunotherapy, Food allergy, Community allergy practice, Discontinuation, Anxiety, Social factors, Pediatrics

## Abstract

**Background:**

Oral immunotherapy (OIT) is an increasingly utilized management strategy for IgE-mediated food allergy. Despite promising efficacy and effectiveness, there is still a lack of data surrounding the reasons for discontinuation of OIT. The primary reason stated in the literature for discontinuation is adverse gastrointestinal effects. Social factors contributing to OIT discontinuation have not been well reported. We hypothesize that social considerations are significant contributors to treatment discontinuation.

**Methods:**

We completed a retrospective chart review of 50 patients treated in community pediatric allergy practices who discontinued OIT out of 507 patients who were started on OIT between October 1, 2017-October 27, 2022. Reasons for discontinuation were identified and classified into five main categories: unsafe care decisions, anxiety, adverse effects of OIT, uncontrolled comorbidity and social factors. Categories were not exclusive.

**Results:**

507 patients were started on OIT**,** with data available for 50 patients who discontinued OIT, aged 10 months to 18 years and 2 months. The overall discontinuation rate was 9.8%, of which 40 patients (80%) discontinued during buildup, 9 patients (18%) discontinued during maintenance and one patient on two food OIT discontinued one food during buildup and one during maintenance (2%). Thirty-four patients (68%) had multiple reasons for discontinuing OIT. Social factors were the most common reason for discontinuation and were identified in 32 patients (64%). Twenty-four patients (48%) discontinued OIT due to adverse effects. Gastrointestinal symptoms were the most prevalent, while anaphylaxis contributed to discontinuation in 15 patients (30%). Anxiety led to discontinuation in 17 patients (34%).

**Conclusions:**

Our data highlights the importance of social factors and anxiety in the success of OIT completion. Our results support the need to consider not only the patient’s medical history, but also their social history and support networks when selecting patients who are good candidates for OIT to optimize the successful completion of OIT.

## Background

Food allergy is common in developed countries, affecting 5–10% of the population [1]. Standard food allergy management involves strict avoidance of allergens and training on managing accidental exposures [2]. The risk of accidental exposure using this strategy is not insignificant, particularly among young children. It is estimated the likelihood of accidental exposure in children is as high as 41% in a 3 year period, with the risk of anaphylaxis in a real-world setting being around 10% [3, 4]. Given the encouraging safety outcomes that have continued to be published for preschool oral immunotherapy, OIT has become increasingly offered both in research and real-world settings in the pediatric population, with multiple OIT-focused guidelines now published [1, 2, 5, 6].

Discontinuation rates for patients on OIT range from 6.7 to 43% [2, 7–13]. The primary reason stated in published studies for discontinuation of OIT is due to gastrointestinal side effects around the time of dosing [5–8, 11, 13–19]. Additional reported factors for discontinuation include repeated allergic reactions with up-dosing, anaphylaxis, eosinophilic esophagitis (EoE), taste aversion/difficulty with dose administration, non-compliance and being lost to follow-up [8, 12, 13, 19–23]. There remains a lack of information surrounding non-medical reasons for treatment discontinuation.

OIT therapy can be a significant burden to the family, as recently described: "Despite the absence of formal medical training, caregivers and patients are expected to act as amateur “healthcare professionals,” assuming multiple medical roles (more typically performed by dietitians, nurses, and physicians) when dealing with the struggles of daily dosing and potential reactions"[24]. Dose administration may be complicated by expected adverse reactions, caregiver and/or patient anxiety around adverse reactions, dose refusal or taste aversion [20]. Caregivers and patients require considerable health literacy and coping skills to navigate these obstacles. One study found that framing non-life-threatening adverse effects positively as a marker of desensitization in patients undergoing OIT reduced family anxiety and improved adherence [25]. Despite these significant social considerations for OIT therapy, analysis of social factors contributing to discontinuation of OIT is underreported in the literature. We hypothesize that social factors play a significant role in the decision to discontinue OIT.

## Methods

We completed a retrospective chart review of 50 patients who discontinued oral immunotherapy between October 1, 2017, and October 27, 2022, in two community pediatric allergy practices in Victoria, BC. Inclusion criteria were any patient who discontinued OIT before October 27, 2022. There was no age limit, although all patients were less than 19 years of age. Patients currently on OIT and patients who had successfully completed OIT were excluded, even if they had previously discontinued OIT. A total of 2 patients were excluded as they had previously discontinued OIT but at the time of data collection had restarted. Patients were started on OIT if they had either: 1) a history of an allergic reaction to the food in question (at home or during optional baseline oral food challenge (OFC) AND either a positive skin prick test (SPT) of ≥ 3 mm, or food-specific immunoglobulin E (s-IgE) of ≥ 0.35 kU/L, OR 2) no history of ingestion of the specific food in question and a s-IgE ≥ 5 kU/L [9]. All patients undergoing OIT underwent dose escalations up to a target maintenance dose of 300 mg food protein.

Information was taken directly from the patient’s chart. The reason for discontinuation was assessed by the clinic nurse, who closely oversaw OIT administration, the patient’s primary allergist (VEC&SBC) and the chart reviewer (AAP). Reasons for discontinuation were divided into five main categories: unsafe care decisions, anxiety, adverse effects of OIT, uncontrolled comorbidity and social factors. The category of social factors was divided into the following subcategories: poor communication between the health care team and patient, dose refusal, caregiver power dynamic, poor health literacy, financial stressors, and difficulty with adherence. All categories were non-exclusive. Patients were placed in categories only if the clinical chart, clinic nurse and primary treating allergist were all in agreement. If there was a discrepancy there was a group discussion with the chart reviewer, clinic nurse and primary treating allergist. In all cases a consensus was reached for which categories the patient was placed in. Every patient who discontinued OIT was placed in at least one category. The categories were not mutually exclusive. The categories were formed following familiarization with the data, and preliminary categories were generated based on insight from multiple team members. Categories were reviewed and defined on an interactive basis until reviewer consensus was achieved, as done in thematic analysis [26]. Table 1 outlines the definitions of each category and subcategory.

**Table 1:** Definition of main categories and subcategories used to classify reasons for which patients discontinued OIT. In all cases, the categories were not exclusive, and individuals could have multiple reasons for discontinuation

Main category	Description of main category
Social factors	Included the following subcategories: • Difficulty with adherence: inability to adhere to the daily OIT dose for any reason • Poor communication between health care team and patient: this included missing appointments without communicating to the clinic, lack of communication with clinic when missing multiple doses at home, or lack of communication following episode of anaphylaxis at home • Dose refusal: difficulty in administration of the daily dose due to child refusing^a^ • Poor health literacy: assessed by the care team based on multiple interactions with the patient and care providers. Health literacy was assessed as a contributing factor if there were persistent gaps in understanding OIT protocols despite multiple information sessions, infographic handouts and written summaries provided to caregivers • Financial stressors: identified as contributing to OIT discontinuation when patient or caregivers noted on intake or during clinical course that appointments were difficult to make due to the financial stress of missing work, the inability to afford equipment necessary for OIT (such as scales and measuring devices) or the inability to afford medications not covered by the provincial health care plan (such as antihistamines).^b^ • Caregiver power dynamic: based on either a caregiver acknowledgment of power imbalance as a reason for unsafe care decisions or witnessed aggression towards child or partner. For example, one caregiver appropriately wishing to administer epinephrine and being overruled by the other caregiver
Adverse effects	Adverse effects of OIT were divided into the following subcategories: • Non-IgE-mediated adverse reactions • IgE-mediated adverse reaction, which were further divided into IgE-mediated symptoms with and without anaphylaxis
Unsafe care decisions	Determined to be a factor for discontinuation if patients demonstrated any of the following: • Unscheduled dose advancement at home • Missing multiple sequential doses without notifying the clinic • Stopping and restarting therapy without notifying the clinic • Missing multiple appointments without notifying the clinic, and repeatedly not responding to communication from the clinic • Inappropriate management of anaphylaxis at home after anaphylaxis education was provided on multiple occasions
Anxiety	Determined to be a factor for discontinuation for patients with any of the following: • Self-identified as anxious • Prior diagnosis of anxiety by a medical professional • Treating allergist and nurse identified as anxious based on recognition of questions, comments or specific voiced concerns that are stereotypical of anxious caregivers and patients
Uncontrolled comorbidity	Included any uncontrolled atopic condition (atopic dermatitis, allergic rhinitis, asthma)

^a^Not all patients that were found to have dose refusal had poor adherence as several caregivers were still able to administer the daily dose

^b^The OIT administration occurred in British Columbia and therefore medical visits were covered by universal health care

Table 2 outlines the definition and examples of the individual responsible for the decision to discontinue treatment. The individual responsible was categorized as the caregiver, the patient, the physician, mutual decision between family and physician, or unknown. All individuals labelled as “patient’s decision” were over the age of 7. The patient’s primary allergist and clinic nurse identified who initiated discontinuation.

**Table 2:** Definitions and examples of how the individual responsible for the decision to discontinue OIT was categorized

Individual responsible	Definition	Example
The patient^a^	The patient decided to discontinue OIT however the physician would have continued to offer OIT. There was no contraindication to continuing OIT	A patient deciding that they have difficulty incorporating their OIT dose into their daily schedule
The caregiver	The caregiver decided to discontinue OIT however the physician would have continued to offer OIT. There was no contraindication to continuing OIT	A caregiver having difficulty with dose administration due to dose refusal
The physician	The physician decided to discontinue OIT however the patient/caregiver wanted to continue OIT	A patient with uncontrolled asthma who wanted to continue OIT despite inadequate asthma control A family up dosing at home without clear instructions from the clinic to do so
Mutual decision between family and physician	After discussion with the physician and caregiver/patient the decision was made to discontinue OIT by both parties	A patient that develops uncontrolled food related anxiety during treatment and both caregiver and physician decide that OIT should be discontinued to focus on anxiety management
Unknown	Unclear if the family discontinued OIT	The patient did not return to the clinic and did not answer communication from the clinic despite multiple attempts to contact them. They were sent a letter explicitly stating they needed to discontinue OIT; however, it is unknown if they truly discontinued OIT

^a^All individuals labelled as “patient’s decision” were over the age of 7

Per the Tri-Council Policy Statement 2 (TCPS2) governing research ethics in Canada, our project fell under quality assurance/quality improvement.

## Results

### Patient characteristics

Patient characteristics are summarized in Table 3. A total of 507 patients were started on OIT from October 1, 2017, to October 27, 2022. Of the 507 patients who started OIT, 50 discontinued OIT, resulting in a discontinuation rate of 9.8%. Of those who discontinued OIT, the median age was 6 years 9 months, but patient ages ranged from 10 months to 18 years and 2 months. Since October 27, 2022, two patients included in this analysis have restarted OIT. Most patients discontinued treatment during buildup phase (80%), while 18% discontinued during maintenance phase. One patient on OIT to multiple foods discontinued one food in buildup and another in maintenance. Most patients were male (66%). Treatment of multiple foods was more highly associated with treatment discontinuation (64% median 2, IQR 1–3.75). Atopic comorbidities were common amongst all patients: 74% had a history of atopic dermatitis, 38% had a history of asthma, 44% had a history of allergic rhinitis, and 22% had a history of an additional food allergy, which was not being managed with OIT. Only 10% of the patients who discontinued had no comorbid condition. Pre-existing anxiety was identified in 28% of patients or caregivers who discontinued: 18% of patients had pre-existing anxiety, 6% had pre-existing anxiety in a caregiver, and 4% had pre-existing anxiety in both patient and caregiver.

**Table 3:** Demographics for the patient population who discontinued OIT between october 1 2017- October 27 2022 (n = 50)

Total number of patients started on OIT from October 1 2017- October 27 2022	507
Total number of patients who discontinued OIT	50 (9.8%)
Gender of patients	
Male	33 (66%)
Female	17 (34%)
Age at start of OIT	Median age 6 years 9 months (10 months-18 years 2 months) IQR 4 years 10 months
Discontinued OIT at buildup	40 (80%)
Discontinued OIT at maintenance	9 (18%)
Discontinued one food at buildup and one food at maintenance	1 (2%)
Number of foods on OIT	
1	18 (36%)
2	10 (20%)
3	9 (18%)
4	6 (12%)
5	3 (6%)
6	1 (2%)
7	1 (2%)
8	2 (4%)
Median	2 (IQR 1–3.75)
Atopic dermatitis	37 (74%)
Asthma at start of OIT^a^	19 (38%)
Allergic rhinitis	22 (44%)
Additional food allergy not on OIT^b^	11 (22%)
Concurrent use of egg or milk ladder	3 (6%) all egg ladder
Other Comorbid Allergic conditions	
FPIES	1 (2%)
Oral allergy syndrome	2 (4%)
Pre-existing EOE	0
No comorbid condition	5 (10%)
Pre-existing Anxiety Patient only Parent only Both	14 (28%) 9 (18%) 3 (6%) 2 (4%)

^a^One additional patient was diagnosed with asthma during OIT. Another patient was diagnosed after stopping OIT. They are not counted in the total number of children with asthma above

^b^4 patients had a history of egg allergy that resolved prior to OIT initiation (3 of these self-resolved and 1 resolved on egg ladder) and 1 child had a flax allergy that resolved prior to OIT initiation

### Reasons for OIT discontinuation

**Fig. 1:**
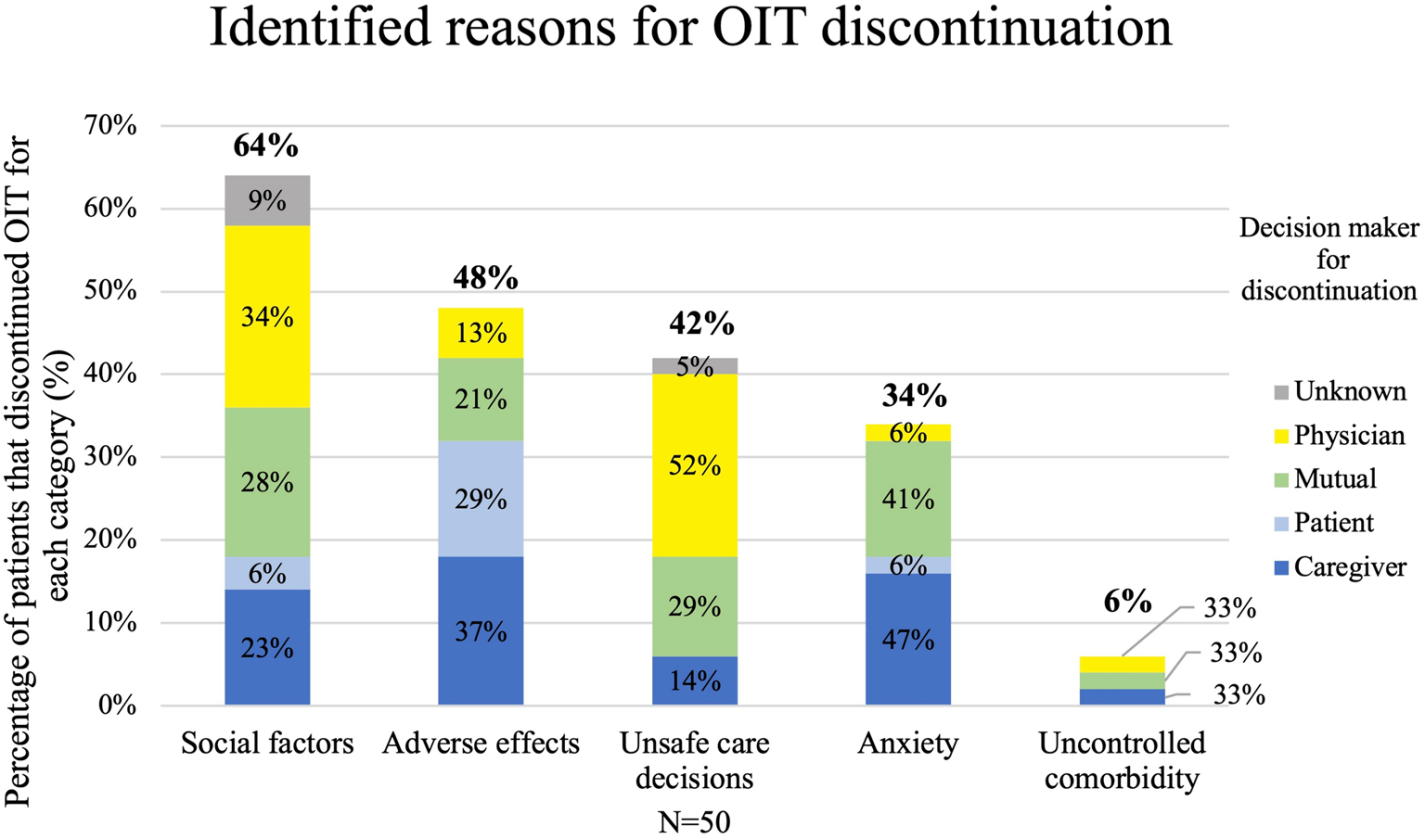
Reasons for discontinuation of OIT were divided into 5 main categories: social factors, adverse effects, unsafe care decisions, anxiety and uncontrolled comorbidity. Adverse effects of OIT include both IgE-mediated (including anaphylaxis) and non-IgE-mediated. Categories were not exclusive. Values are expressed as a percentage of all patients who discontinued OIT (n = 50)

Figure 1 shows the main reasons for discontinuation based on 5 main categories: social factors, adverse effects, unsafe care decisions, anxiety, and uncontrolled comorbidity. Adverse effects of OIT include both IgE-mediated (including anaphylaxis) and non-IgE-mediated symptoms. Categories were not exclusive.

Most patients (64%) discontinued treatment secondary to social factors, 48% discontinued due to adverse effects of OIT, 42% discontinued related to unsafe care decisions, 34% had anxiety contribute to discontinuation, and 6% secondary to uncontrolled comorbidities (Fig. 1). The decision to discontinue was initiated by the caregiver in 32% of cases, 26% were discontinued as a mutual decision between caregivers and physician, 22% were instructed by the physician to discontinue, 14% initiated discontinuation themselves. In 6% families ceased contact with the clinic and were sent letters to discontinue by the physician.

Of the three patients who discontinued due to comorbidities, two patients were advised to discontinue due to poorly controlled asthma with poor medication compliance, and one patient discontinued for atopic dermatitis as caregivers wished to focus on atopic dermatitis management.

**Fig. 2:**
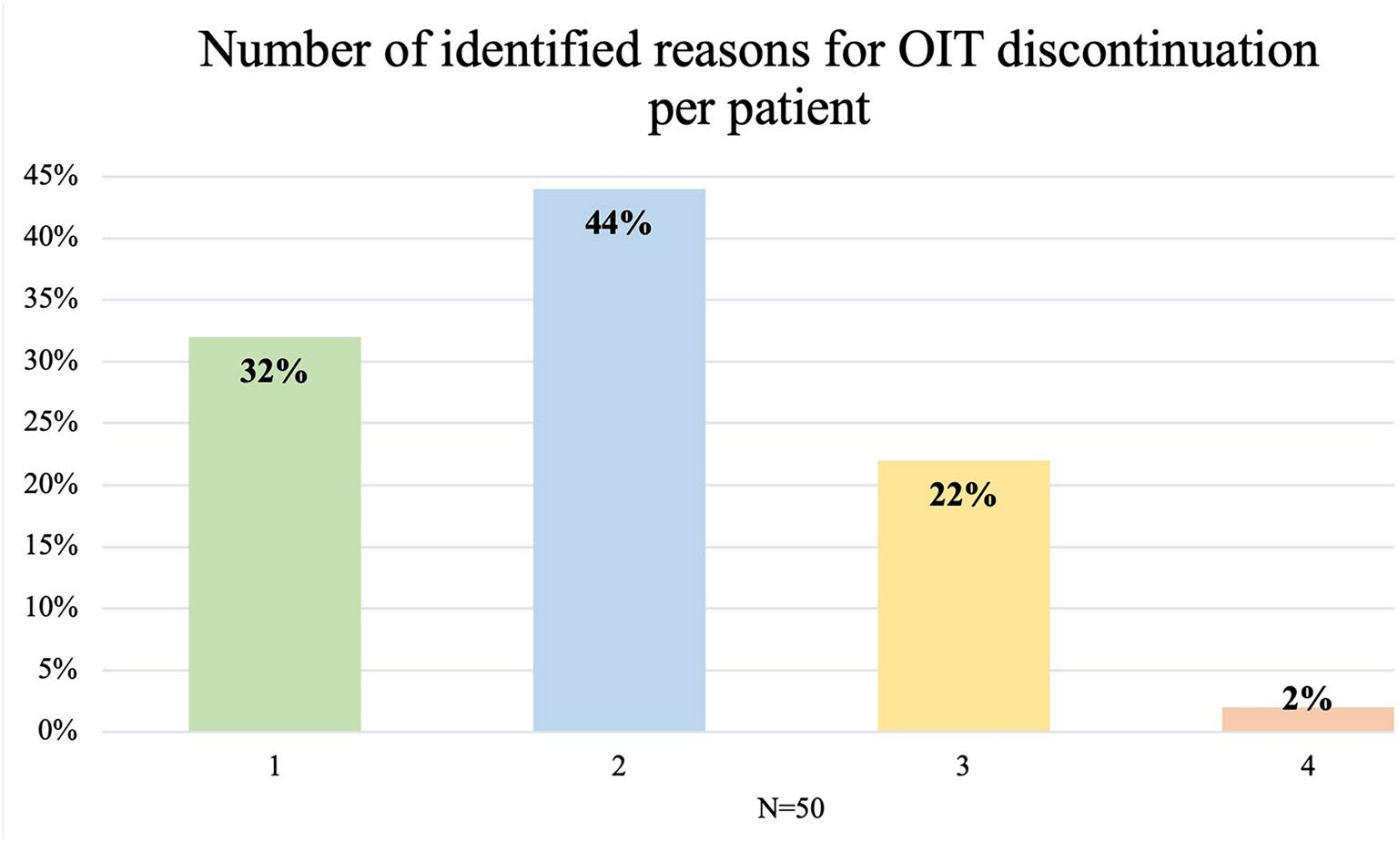
Number of identified reasons for OIT discontinuation for each patient, expressed as a percentage of all patients that discontinued OIT

Most patients had multiple reasons for discontinuation (68%). Only 32% of patients were identified as having 1 main category as a reason for discontinuation, 44% had two categories as the reason for discontinuation, 22% 3 main categories and 2% had 4 reasons for discontinuation (Fig. 2).

### Social reasons for OIT discontinuation

**Fig. 3:**
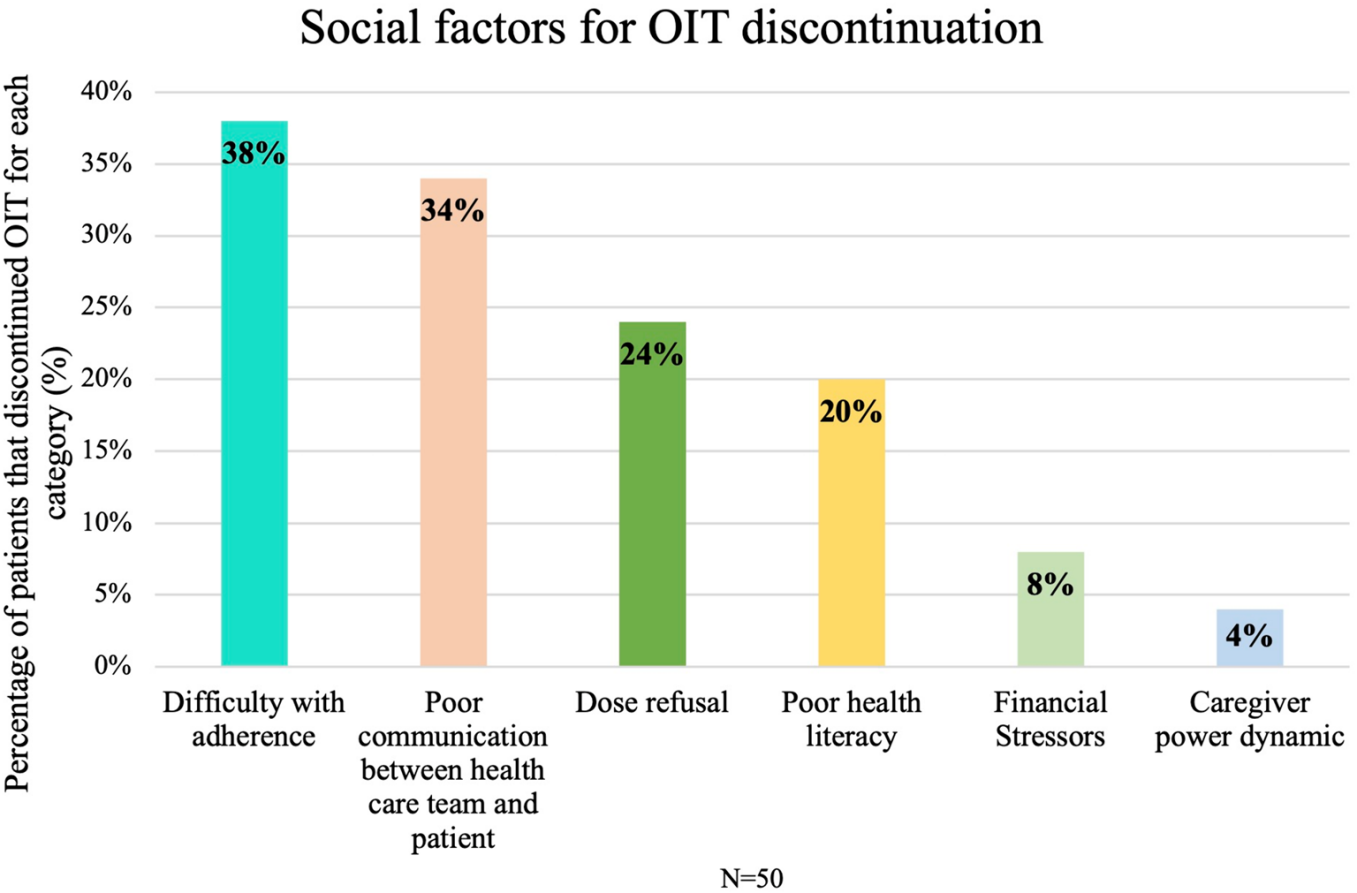
Social factors for OIT discontinuation were divided into the following non-exclusive subcategories: poor communication between health care team and patient, dose refusal, caregiver power dynamic, poor health literacy, financial stressors, and difficulty with adherence. 32 individuals were identified as having social reasons that contributed to OIT discontinuation

Figure 3 demonstrates the social reasons for discontinuing OIT based on 6 subcategories: poor communication between the health care team and patient/caregiver, dose refusal, caregiver power dynamic, poor health literacy, financial stressors, and difficulty with adherence. Categories were not exclusive. Thirty-two out of the 50 individuals (64%) who discontinued OIT were identified as having social reasons contributing to OIT discontinuation. Thirty- eight percent of the patients who discontinued due to social reasons had difficulty with daily dose administration, 34% had poor communication between the health care team and patient/caregiver, 24% had dose refusal, 20% had poor health literacy, 8% experienced financial stressors leading to discontinuation and 4% had a challenging caregiver power dynamic resulting in discontinuation (Fig. 3).

### Adverse side effects as the reason for OIT discontinuation

**Fig. 4:**
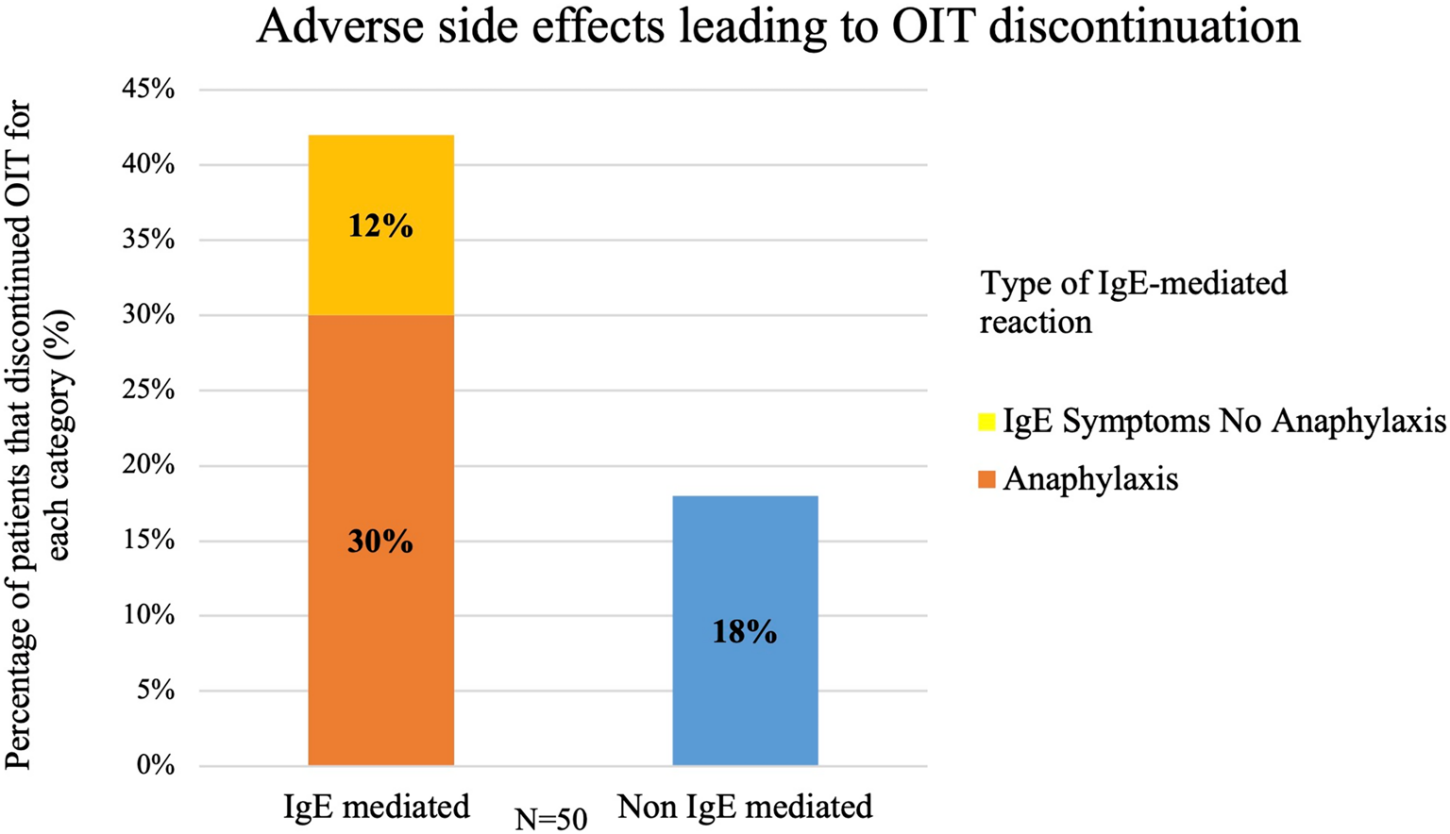
Adverse side effects of OIT divided into IgE-mediated, anaphylaxis and non-IgE-mediated. Each patient could have experienced multiple adverse side effects; categories were not exclusive. A total of 24 patients were identified as having adverse side effects that contributed to the discontinuation of OIT

Figure 4 illustrates adverse side effects as reasons for OIT discontinuation divided into IgE-mediated, anaphylaxis and non-IgE-mediated. Categories were not exclusive. A total of 24 out of 50 patients who discontinued (48%) were identified as having adverse side effects that contribute to OIT discontinuation. Thirty percent of the total group who discontinued OIT had anaphylaxis identified as a reason for OIT discontinuation, 12% had IgE-mediated symptoms without anaphylaxis identified as a reason for OIT discontinuation, and 18% had non-IgE-mediated symptoms without anaphylaxis identified as a reason for OIT discontinuation. Patients who had non-IgE-mediated reactions leading to discontinuation of OIT experienced daily symptoms, and they were all gastrointestinal-related. All patients (n = 9) with non-IgE-mediated symptoms had complete resolution of symptoms following OIT discontinuation.

### Anxiety as the reason for OIT discontinuation

**Fig. 5:**
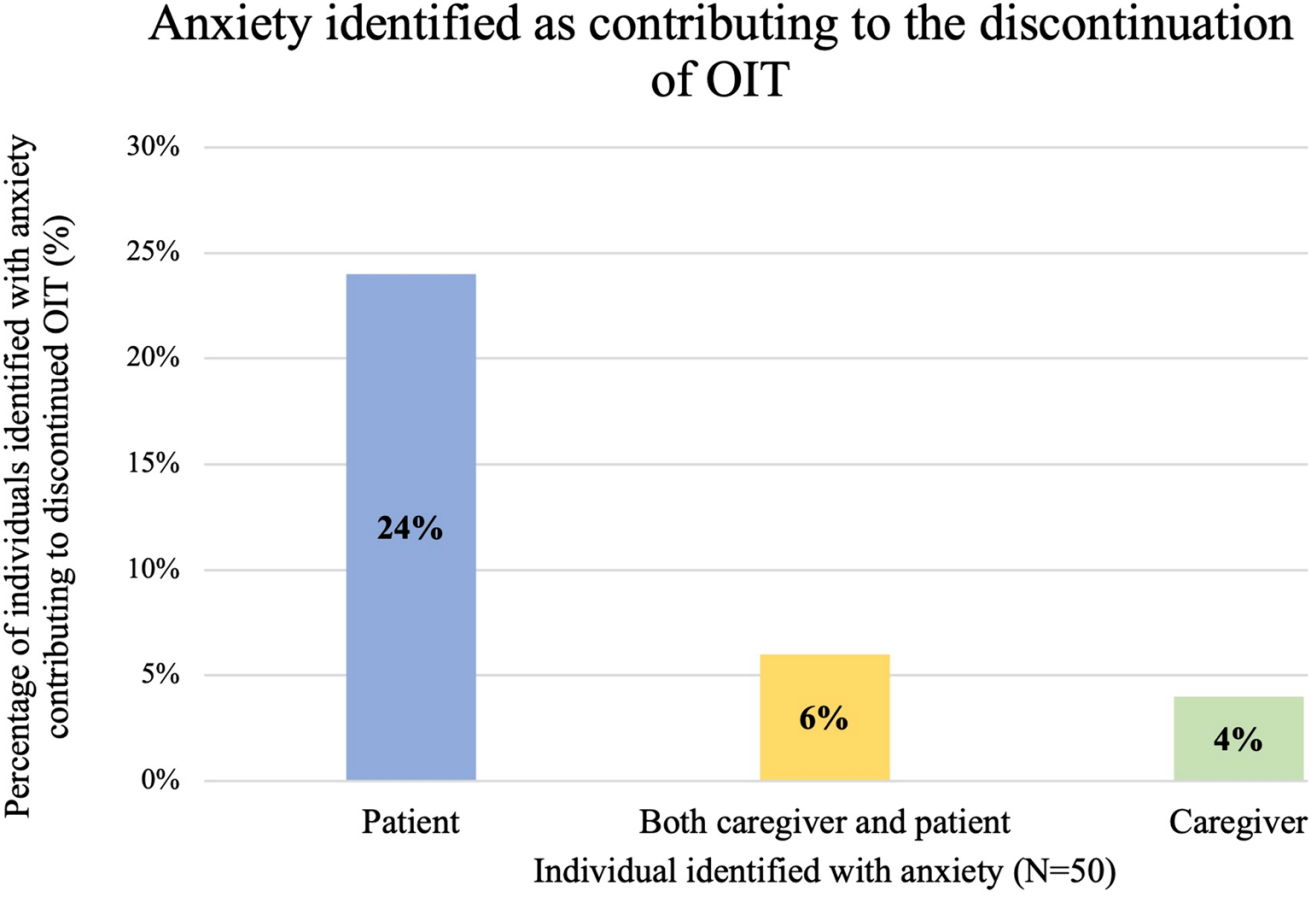
Anxiety identified as contributing to the discontinuation of OIT divided into patient anxiety, caregiver anxiety or both. A total of 17 patients were identified as having anxiety that contributed to the discontinuation of OIT

Out of the 50 patients who discontinued OIT, 17 (34%) had anxiety identified as a reason for discontinuation (Fig. 5). The individual experiencing anxiety was most commonly the patient (24%) but could also be both patient and caregiver (6%) or the caregiver alone (4%). Of the 17 cases where anxiety was identified as a contributing factor in OIT discontinuation, 65% had been pre-identified as anxious and 45% of the pre-identified families had been referred to a mental health professional for counselling.

## Discussion

This is the first real-world retrospective study that focuses on the physician's perspective for reasons for OIT discontinuation. The overall rate of discontinuation in our review was 9.8%. Discontinuation rates range in the literature from 6.7% to as high as 43% with most studies quoting between a 10–20% drop out rate [7–9, 12, 14, 27]. Only two patients who were included in this chart review have since restarted OIT, indicating that for most patients who discontinue OIT, it is a long-term decision.

Social reasons for discontinuation have not been directly addressed in most clinical trials. Vickery et al., mentioned in one of their figures that 5/80 discontinued patients did so for “other reasons” which included scheduling conflicts, difficulty with time commitment and parental concerns [14]. Bird et al. also mentions that 2/6 of their participants discontinued due to anxiety and compliance concerns [10]. These were both clinical trials which likely self-selected for individuals who were highly motivated and less likely to have challenges with social factors. In the Goldberg et al. real-world single center study they discuss that 2/6 patients discontinued due to difficulty incorporating daily dosing into their routine, however, this was a small study with only 60 patients total on OIT and did not focus on reasons for discontinuation [12]. Our study is the first to our knowledge to evaluate the social factors in a clinical setting that contribute to discontinuation of OIT. Importantly, social factors contributed to OIT discontinuation in over half of patients (Fig. 1). A recent international Delphi panel advised that patients should be prepared for the possibility of discontinuation before starting OIT as part of the counselling process. Many of the reasons cited by this panel included social reasons [28]. This speaks to the importance of clinicians asking about social factors and recognizing them as significant contributors to discontinuation [20].

Our results demonstrate that anxiety is a contributor to discontinuation of OIT in one-third of patients (Fig. 1). It has been reported that anxiety occurs at a higher rate in both caregivers and children with food allergy compared to the general population [20, 30, 31]. Protudjer et al. reported that maternal anxiety occurred in 34% of mothers of children with food allergy and 44% of children with food allergy during the coronavirus pandemic [30]. Anxiety is an important consideration in OIT administration, and it is our experience that many children and caregivers have anxiety around up-dosing and the potential for anaphylaxis. In centers that offer psychologic support to all patients undergoing OIT, 66% utilized these supports, with the predominant reason being mood and anxiety symptoms related to OIT [29]. Additionally, anxiety can manifest in gastrointestinal symptoms, which can make it challenging to interpret whether gastrointestinal symptoms are an adverse effect of OIT or a symptom of uncontrolled anxiety. This is particularly true for younger children who do not necessarily have the vocabulary to articulate the anxiety they are experiencing [32]. Given the effect anxiety can have on a patient’s success in OIT, we recommend anxiety screening before OIT initiation and at subsequent visits. Knibb et al. found that in Canada only 13.9% of caregivers were screened for food related anxiety and 9.7% of children. Questionnaires that could be used include Scale of Food Allergy Anxiety (SOFAA) for children 8 and up and Impairment Measure for Parental Food Allergy-associated Anxiety and Coping Tool (IMPAACT) for parents [33, 34]. There is currently a lack of standardized screening tools for food related anxiety in children less than 8. If anxiety is identified, then prompt management may help increase a patient’s success in OIT completion. Our clinics now refer for counselling prior to initiating OIT if anxiety is identified. This approach is consistent with recent recommendations regarding patient preparation for OIT [28]. Polloni et al. described that anxiety, mood disorders, increased distress and excessive worry/fear can affect compliance and the ability to progress through therapy and when addressed and treated appropriately all patients reported a moderate to great improvement in their situation [29].

Over two-thirds of the patients (68%) had multiple reasons for discontinuation of treatment (Fig. 2). This reflects the impact of OIT on multiple aspects of family life [24], including the burden of daily dosing and regular physician appointments, anxiety around up-dosing and potential for anaphylaxis, restriction of physical activities around the time of dosing, OIT-mediated side effects and potential for financial burden. These considerations become particularly important when evaluating adolescents, as recurrent adverse reactions can lead to missed days at school, and exercise limitations can affect their social life with the limitations to participation in extracurricular sports [8]. Families must be informed of the treatment burden before initiating OIT, and ongoing shared decision-making continues throughout the course of treatment [28]. There may be times where the goals and priorities of caregivers are not aligned with those of the adolescent. It is the clinician’s role to advocate for the patient and ensure adolescents are active decision makers in their care, whether this be continuing with OIT or even if this means discontinuing OIT [24]. Physicians need to be able to support the patient without instilling feelings of failure.

Much of the current literature reports adverse effects as the primary reason for OIT discontinuation, of which GI symptoms are the most common [6, 8, 16]. This is similar in our group of patients; 48% discontinued due to adverse side effects of OIT, with the most common side effect being gastrointestinal (Fig. 4). This is slightly lower than reported in the literature, with rates that range from 58% to 72% [8, 10, 11]. Our center did use a standardized protocol [35] to address gastrointestinal symptoms while on OIT, which may have contributed to the lower discontinuation rate due to gastrointestinal side effects.

The rate of anaphylaxis among patients who discontinued OIT was 32%, with 16 of the 50 patients experiencing anaphylaxis at some point during OIT. Half of the episodes of anaphylaxis were associated with a cofactor, the most common being exercise around the time of dose administration. There is literature that shows a meaningful number of families and patients experience acute distress after food-induced anaphylaxis and that the use of epinephrine is significantly associated with OIT discontinuation [29, 35]. In Polloni et al. they highlight the importance of offering psychological support to families and patients who have experienced anaphylaxis during OIT [29]. This data from Pollini et al., along with our own data, has led us to proactively suggest psychosocial support, and includes the use of a patient handout, when families report OIT associated anaphylaxis [29]. Additionally, while we try to mitigate the risk of anaphylaxis by reducing patient exposures to cofactors, we recognize this can lead to an increased treatment burden [9, 24]. For example, it can become difficult for an active adolescent with extracurriculars to schedule daily dosing around extracurricular sports and social events.

This study aimed to identify reasons for OIT discontinuation in the real world with the eventual goal of better identifying patients at risk of OIT discontinuation. Our data highlights a novel understanding of the importance of social factors and anxiety in the success of OIT completion (Figs. 1, 3 and 5). Difficulty with adherence and poor communication were the most frequently identified social factors leading to discontinuation (Fig. 3). A detailed social history may help identify patients at risk of OIT discontinuation and may allow for targeted interventions and support. Early identification of challenges with adherence can lead to prompt interventions such as referral to dieticians for picky eaters and providing a written management plan for alternative dosing options. Regular and timely follow-up is also important for families who are identified as at risk for discontinuation, particularly those with poor communication. Education around the time commitment and burden of the scheduling of OIT is essential in ensuring families are well informed on OIT before initiation. Fleischer and Greenhawt have outlined a checklist for shared decision-making conversations and a list of common logistical challenges that families face with OIT. This can be used to guide conversations to help patients and families make an educated decision about which treatment strategy is best given their family’s capacity [36].

During OIT, there should be regular appointments with questions focusing on barriers to dose administration, anxiety around dosing and the practicality of dose administration. It can be an exceptional challenge for many families to ensure a toddler reliably takes the full dose or for a teenager to find time between physical activity to take their dose. Our data is consistent with previously published results showing that gastrointestinal side effects are very common during OIT, and if not managed promptly, can contribute to discontinuation [14, 27]. It was our experience that OIT-eager caregivers often downplayed GI symptoms, thus highlighting the importance of asking the patient, rather than caregiver, about symptoms so they can be systematically managed [37].

Our results demonstrate that OIT may not be an appropriate treatment option for all patients [2]. OIT can have a significant treatment burden. For families where OIT is not improving their quality of life, it is important to recognize that strict avoidance continues to be a very reasonable option for the management of food allergy.

Our study has several limitations, including a retrospective, single-center design. Assessment of anxiety in patients and caregivers was based on allergist and pediatric OIT nurse interaction and assessment or patient self-identification rather than a formal screening tool, which has potential biases. We tried to mitigate these biases by collecting data from several sources, such as the chart, the clinic’s nurse clinician and the treating allergist. We only placed patients in specific categories if all three sources agreed. Additionally, the primary chart reviewer did not have a therapeutic relationship with the families included in this study to enhance objectivity. We did not evaluate social factors or anxiety in the group who successfully completed OIT. As such, a comparison of the effect size of social barriers and anxiety on the success of OIT was not possible.

Further OIT studies should include data on reasons for discontinuation, specifically social and mental health categories, and logistical challenges for families. Patient questionnaires and a standardized form filled out by allergists to identify known reasons for discontinuation may help determine what supportive measures best improve OIT success.

OIT provides families with an alternative management strategy for IgE-mediated food allergy, but it does come with its own burden of treatment. For OIT to be successful, it is essential that we continue to engage with families and maintain an open line of communication during OIT treatment to better support them not only regarding adverse side effects but also their psychosocial needs.

## Data Availability

Data is provided within the manuscript or supplementary information files.

## Abbreviations

OIT: Oral immunotherapy
FPIES: Food protein induced enterocolitis syndrome
EOE: Eosinophilic esophagitis
OFC: Oral food challenge
SPT: Skin prick test
TCPS2: Tri council policy statement 2
GI: Gastrointestinal
